# Altered Brain Functional Connectome in Migraine with and without Restless Legs Syndrome: A Resting-State Functional MRI Study

**DOI:** 10.3389/fneur.2018.00025

**Published:** 2018-01-30

**Authors:** Fu-Chi Yang, Kun-Hsien Chou, Ai-Ling Hsu, Jong-Ling Fuh, Jiing-Feng Lirng, Hung-Wen Kao, Ching-Po Lin, Shuu-Jiun Wang

**Affiliations:** ^1^Department of Neurology, Tri-Service General Hospital, National Defense Medical Center, Taipei, Taiwan; ^2^Institute of Brain Science, National Yang-Ming University, Taipei, Taiwan; ^3^Institute of Neuroscience, National Yang-Ming University, Taipei, Taiwan; ^4^Brain Research Center, National Yang-Ming University, Taipei, Taiwan; ^5^Graduate Institute of Biomedical Electronics and Bioinformatics, National Taiwan University, Taipei, Taiwan; ^6^Faculty of Medicine, National Yang-Ming University School of Medicine, Taipei, Taiwan; ^7^Neurological Institute, Taipei Veterans General Hospital, Taipei, Taiwan; ^8^Department of Radiology, National Yang-Ming University, Taipei, Taiwan; ^9^Department of Radiology, Taipei Veterans General Hospital, Taipei, Taiwan; ^10^Department of Radiology, Tri-Service General Hospital, National Defense Medical Center, Taipei, Taiwan; ^11^Department of Biomedical Imaging and Radiological Sciences, National Yang-Ming University, Taipei, Taiwan

**Keywords:** MRI, migraine, restless legs syndrome, functional connectivity, connectome

## Abstract

**Background:**

Migraine is frequently comorbid with restless legs syndrome (RLS), both displaying functional connectivity (FC) alterations in multiple brain networks, although the neurological basis of this association is unknown.

**Methods:**

We performed resting-state functional magnetic resonance imaging and network-wise analysis of FC in migraine patients with and without RLS and healthy controls (CRL). Network-based statistics (NBS) and composite FC matrix analyses were performed to identify the patterns of FC changes. Correlation analyses were performed to identify associations between alterations in FC and clinical profiles.

**Results:**

NBS results revealed that both migraine patients with and without RLS exhibited lower FC than CRL in the dorsal attention, salience, default mode, cingulo-opercular, visual, frontoparietal, auditory, and sensory/somatomotor networks. Further composite FC matrix analyses revealed differences in FC of the salience, default mode to subcortical and frontoparietal, auditory to salience, and memory retrieval networks between migraine patients with and without RLS. There was a trend toward a negative association between RLS severity and cross-network abnormalities in the default mode to subcortical network.

**Discussion:**

Migraine patients with and without RLS exhibit disruptions of brain FC. Such findings suggest that these disorders are associated with differential neuropathological mechanisms and may aid in the future development of neuroimaging-driven biomarkers for these conditions.

## Introduction

Migraine is a primary headache disorder affecting 10–20% of the general population, which occurs predominantly in women (female-to-male ratio, 2–3:1). It is characterized by recurrent headaches associated with moderate-to-severe pulsating pain and sensory hypersensitivity (1). Accumulating evidence suggests that restless legs syndrome (RLS) is a common comorbidity in patients with migraine (2–10). Furthermore, it has been reported that periodic limb movement disorders (including RLS) may influence the clinical presentation, severity, frequency, and treatment efficacy of migraine in children (6). RLS is a sensorimotor disorder characterized by a deeply unpleasant sensation in the legs when at rest, especially at or near bedtime, and motor restlessness that can only be relieved by voluntary movement. The prevalence of RLS in patients with migraine is higher than that in the general population (5, 10). Although some researchers have proposed that migraine and RLS share pathophysiological characteristics, such as abnormalities in dopaminergic or iron systems as well as genetic variations (2, 11), the neural mechanisms governing the comorbidity of migraine and RLS remain largely unknown.

Recent evidence suggests that disruption of coordinated activity between and within brain networks may be involved in the pathogenesis of various neuropsychological disorders (12). Resting-state functional magnetic resonance imaging (rs-fMRI) studies have suggested that the pathophysiology of migraine is associated with altered functional connectivity (FC) in several brain networks, such as the default mode network (DMN), visual, executive control, attention, and salience networks. These functional disturbances may also relate to the sensory, affective, and cognitive components of pain processing in migraine patients (13–15). In contrast, the presence of functional abnormalities in patients with RLS remains controversial. Previous studies have reported dysfunction of multiple brain networks such as the thalamic, sensory-motor, limbic/nociceptive networks, and the DMN in patients with RLS (16–19). Although previous evidence supports the existence of functional network disturbances in patients with migraine and RLS, it remains unknown whether these disturbances are associated with the comorbidity of migraine and RLS. Furthermore, most previous studies of migraine and RLS have examined connectivity with a single seed region or *via* pairwise coupling, which may fail to elucidate the role of such connections within the greater network. Moreover, these methods do not provide insight into how migraine with or without RLS (MIG w or w/o RLS) is associated with the restructuring of functional networks, particularly at the connectome level. In contrast, network-based statistics (NBS) provide a general framework for the characterization of specific network components, which may help identify whether and which subnetworks exhibit significant functional differences between patients with MIG w or w/o RLS (20). Such an understanding of the patterns of FC alterations between and within networks may aid in elucidating the neuropathological mechanisms underlying the comorbidity of migraine and RLS.

Therefore, to address these issues, we performed a comprehensive investigation of the whole-brain functional connectome to identify the patterns of FC changes in migraine patients with and without RLS. Using rs-fMRI, we aimed to demonstrate the association between connectivity patterns and diagnosis in such patients and to examine the following hypotheses: (1) brain FC is altered in migraine patients with or without RLS when compared to controls; (2) migraine patients with or without RLS show changes in FC patterns; and (3) changes in FC correlate with clinical variables.

## Materials and Methods

### Participants

The present study was approved by the Ethics Committee of Taipei Veterans General Hospital (TVGH) and Tri-Service General Hospital (TSGH) in Taiwan. All methods were carried out in accordance with the approved guidelines. All participants signed a written informed consent form after a full written and verbal explanation of the study. Patients with episodic migraine were recruited from the Headache Clinic at TVGH and TSGH. All participants met the diagnostic criteria for episodic migraine based on the third edition of the International Classification of Headache Disorders (21). None had concomitant primary or secondary headache disorders. Patients were further classified based on the presence or absence of a diagnosis of primary RLS, which was made in accordance with criteria outlined by the International Restless Legs Syndrome Study Group (IRLSSG) (22).

All patients underwent a detailed history, physical examination, laboratory testing, and electromyography to exclude secondary forms of RLS. No patients had a history of psychiatric symptoms, and none were treated with psychotropic medications, except for two patients with MIG w RLS taking a dopaminergic agent (ropinirole). Other clinical data such as sex, age, duration of RLS, duration of migraine, and sleep quality were recorded. RLS severity was assessed using the IRLSSG rating scale (23). Patients were asked to stop taking migraine-preventive medications (B-blocker or flunarizine) at least 2 days prior to imaging. All patients were symptoms free (migraine or RLS) for at least 2 days before the imaging scan. No acute migraine or RLS attacks occurred during the scanning sessions.

The healthy control (CRL) group was composed of volunteers without RLS or migraine matched for age, sex, and handedness. Control participants were recruited through community advertisements and hospital patient pools. Exclusion criteria for control participants included a family history of RLS or migraine, prior diagnosis of a primary or secondary headache disorder, and any chronic pain condition. Statistical analysis of demographic data was performed using SPSS software version 20 (SPSS, Chicago, IL, USA). Our study included 22 patients with migraine without idiopathic RLS (3 men, 19 women; mean age: 32.4 years; and age range: 20–54 years) and 22 patients with migraine with idiopathic RLS (2 men, 20 women; mean age: 33.0 years; and age range: 21–51 years). The control group was composed of 19 age- and sex-matched adults (2 men, 17 women; mean age: 33.3 years; and age range: 23–54 years) with no history of neurologic or psychiatric disease.

### Data Collection

All images were collected at TVGH using a 3T MRI scanner (Discovery MR750; General Electric Healthcare, Milwaukee, WI, USA) equipped with an eight-channel phase array head coil. A whole-brain axial T1-weighted anatomical scan was acquired using a three-dimensional inversion recovery prepared fast spoiled gradient recalled sequence [repetition time (TR) = 9.2 ms, echo time (TE) = 3.7 ms, inversion time = 450 ms, flip angle = 12°, number of excitations (NEX) = 1, field-of-view (FOV) = 256 mm × 256 mm, 1 mm isotropic resolution, and 172 slices without interslice gap]. Whole-brain spontaneous activity was measured using a T2*-weighted blood oxygenation level-dependent contrast-sensitive gradient-echo echo-planar imaging sequence (TR = 2,500 ms, TE = 30 ms, flip angle = 90°, NEX = 1, FOV = 222 mm × 222 mm, voxel size = 3.47 mm × 3.47 mm × 3.5 mm, and 43 interleaved axial slices without interslice gap). Two-hundred image volumes were acquired for each participant. During the rs-fMRI acquisition, the participants were instructed to remain relaxed and awake with their eyes closed. An experienced radiologist evaluated each brain anatomical scan for gross abnormalities.

### Data Preprocessing of rs-fMRI

Data preprocessing was conducted using AFNI (Analysis of Functional Neuroimages^1^), FSL v.5.0.9 (Functional MRI of the Brain Software Library^2^), and SPM8 (Statistical Parametric Mapping software^3^). All functional images were first preprocessed in the original native space and then spatially normalized into the standard Montreal Neurological Institute (MNI) space for further brain functional network analysis. Prior to the preprocessing, the first 10 volumes of the acquired rs-fMRI dataset were discarded for magnetic homogenization. After removing the unwanted volumes, every individual data set underwent the following standard preprocessing: head movement correction (MCFLIRT), slice-timing correction (slicetimer), large transient and polynomial trend removal (3dDespike and 3dDetrend), non-brain tissue removal (BET), and spatial smoothing (Gaussian kernel with a 6-mm full-width at half-maximum, fslmaths). None of the participants were excluded based on the exclusion criterion of a maximum displacement larger than 2 mm and a rotation greater than 2° in any direction. To further minimize the potential contributions of non-neuronal physiological fluctuations, cardiac and respiration profiles were estimated by motion-corrected time series data using the PESTICA toolbox (24). Finally, band-pass filtering (0.01–0.08 Hz) and nuisance signal regression were combined into a single regression step (3dTproject) to remove the confounding signals from the time course of each voxel. The nuisance signals in the current study were modeled by an averaging cerebrospinal fluid time series, a local whiter matter time series (25), the Friston 24-motion-parameter model, and estimated cardiac and respiration fluctuations. The mean global signal was not included among the nuisance variables to avoid artificially induced anti-correlation patterns of brain functional networks (26). After completing the abovementioned preprocessing pipeline, the resulting rs-fMRI data sets were registered into standard MNI space using an EPI template (spm_normalise) and then resampled to a 2-mm isotropic resolution.

### Motion-Related Analysis of rs-fMRI Data Set

For motion-related issues of rs-fMRI, we not only performed the subject-level motion control but also managed group-level micromovements by additionally modeling the individual mean framewise displacements (FD, calculated by fsl_motion_outliers). According to the FD exclusion criteria, subjects with the following two conditions were acceptable to be further excluded: (1) mean FD was larger than 0.25 mm, and (2) over 40% of the image volume was removed after censoring all time points with FDs > 0.25 mm (27). Combining with Friston 24-motion regressors in the subject-level nuisance regression, this two-level regression approach and conservative exclusion criteria for head motion minimized the potential influence of motion on both subject-level connectivity estimation and the group-level results interpretation.

### Node and Edge Definitions in Brain Functional Networks

**Figure 1:**
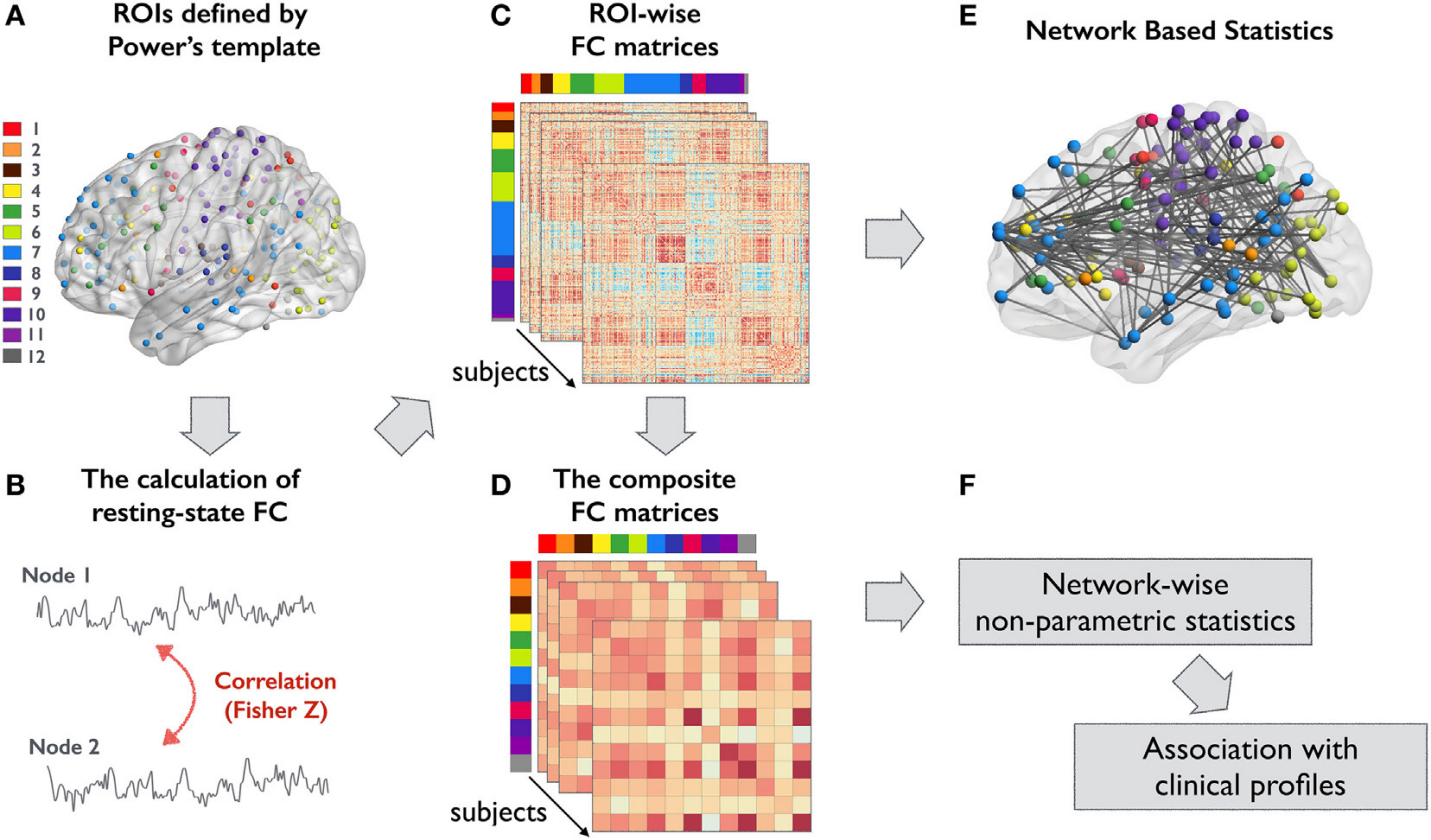
Overview of the analytical steps in this study. **(A)** ROIs defined by Power’s template were used to form ROI-wise FC matrix for all participants. Each color represents a different large-scale functional subsystem: 1, dorsal attention; 2, ventral attention; 3, subcortical; 4, salience; 5, frontoparietal task control; 6, visual; 7, default mode; 8, auditory; 9, cingulo-opercular task control; 10, sensory/somatomotor; 11, memory; and 12, cerebellar network **(B)** FC between an ROI pair was computed using Pearson correlation coefficient and then transformed to *z*-value. **(C)** An example of a connectivity matrix from a single subject. **(D)** Composite *z*-score approach was used to integrate the high-resolution connectivity matrices into 12 functional subsystems. **(E,F)** The corresponding statistical approaches for analyzing **(C,D)**, respectively. Abbreviations: FC, functional connectivity; ROIs, regions of interest.

The analytical steps of the present study are summarized in Figure 1. To investigate FC differences among groups, we utilized a scheme that integrates fMRI meta-analysis findings using MNI coordinates to define nodes within large-scale networks (28). The edges were estimated based on the statistical dependence between nodes in residual time series data. Specifically, these nodes were subdivided into 12 large-scale functional subsystems across cortical and subcortical areas: dorsal attention, ventral attention, subcortical, salience, frontoparietal, visual, default mode, auditory, cingulo-opercular, somatomotor, memory retrieval, and cerebellar networks. (Coordinates of the 236 nodes are listed in Table S1 in Supplementary Material.) A 10-mm-diameter sphere centered at each coordinate represented each node and was used to extract the corresponding mean time series data. A 236 × 236 inter-regional FC matrix was constructed for each participant by calculating the Pearson correlation coefficient between all pairs of nodes. Finally, Fisher’s *r*-to-*z* transformation was used to improve the normality of the correlation estimates. Then, the unthresholded FC matrices (including both positive and negative *z*-values) were used for further network-level analysis.

### Analysis of Whole-Brain FC Changes Using NBS

We performed NBS with analyses of covariance to identify network-wise FC changes (20). Specifically, the primary cluster-defining threshold (*t* > 4, *p* < 5 × 10^−4^, one-tailed) was first applied to identify a set of suprathreshold edges, at which time all connected components and their sizes were determined. Five-thousand permutations were then used to generate the empirical null distribution for the size of the largest connected component and to estimate the significance of each identified component. Only components whose size exceeded the family-wise error (FWE)-corrected *p*-value of <0.05 were deemed to be statistically significant. Participant age, sex, and mean FD were regarded as nuisance variables. Since NBS results are highly dependent on the initial cluster-defining threshold, we performed an additional series of NBS analyses with different initial cluster-defining thresholds (Figure S1 in Supplementary Material). Because NBS aims to identify network-level FC changes across the whole brain, we then performed further pairwise connectivity analyses to localize the connectivity changes within or between brain functional subsystems.

### Pairwise Connectivity Analysis of Brain Functional Subsystems

To localize the FC changes within and between the 12 functional subsystems, the individual 236 × 236 interregional FC matrix was integrated into a 12 × 12 FC matrix using composite *z*-score approach. The composite *z*-score was calculated by averaging the *z*-values of all nodes residing in the corresponding network pairs. These integrated matrices represent the intra- and inter-FC strengths of the 12 functional subsystems. A non-parametric statistical analysis with 5,000 permutations and the same statistical design was used to identify between-group differences in the aggregated FC matrices. The threshold of significance was set at a false discovery rate (FDR)-corrected *p*-value of <0.05. We further performed a conjunction analysis to identify the shared intra- and interconnectivity changes in functional subsystems in each patient group. This analysis was performed by searching for the union-section of the FDR-thresholded matrices from patient–control comparisons (29). Effect size was computed using Cohen’s *d* for each statistical comparison (30).

### Correlations of Connectivity Measures with Clinical Profiles

Spearman’s rank correlation analyses with nuisance covariates (age, sex, and mean FD) were used to identify associations between the strengths of abnormal connections and clinical variables (duration of migraine, headache frequency, duration of RLS, IRLSSG severity scale, Pittsburgh Sleep Quality Index, Hospital Anxiety and Depression Scale, and Beck Depression Inventory score). Only connections identified using composite FC matrix analysis for both patient groups were included for correlation analyses.

## Results

### Patient’s Characteristics and rs-fMRI Motion Profiles

There were no significant differences in sex (*p* = 0.89) or age (*p* = 0.92) between patients and control participants. Demographic and clinical characteristics are summarized in Table 1. During the rs-fMRI scans, no participants exhibited maximum rotations ≥ 2.0° or displacements ≥ 2.0 mm in any direction. No significant differences in mean FD were observed between patients and controls (*p* = 0.88).

**Table 1:** Demographic and clinical characteristics.

	MIG w RLS (*n* = 22)	MIG w/o RLS (*n* = 22)	CRL (*n* = 19)	*p*-Value
Age	33.0 ± 9.0	32.4 ± 7.7	33.5 ± 9.1	0.92
Sex (male/female)	2/20	3/19	2/17	0.89
Aura/no aura	11/11	11/11	/	/
Mean migraine duration (years)	14.5 ± 10.8	11.9 ± 7.4	/	0.38
Mean migraine frequency (days/month)	7.8 ± 6.3	6.1 ± 5.8	/	0.42
Mean RLS duration (months)	49.8 ± 44.1	/	/	/
Mean IRLSSG severity score (0–40)	10.6 ± 8.5	/	/	/
Mean PSQI total score (0–21)	10.5 ± 4.0	7.9 ± 3.1	/	0.02
Mean HADS score (0–42)	13.7 ± 6.0	12.5 ± 7.1	/	0.56
Mean BDI score (0–63)	9.1 ± 5.3	8.4 ± 5.9	/	0.67
Frame displacement (mm)	0.08 ± 0.04	0.07 ± 0.03	0.08 ± 0.02	0.88

*BDI, Beck Depression Inventory; CRL, healthy controls; HADS, Hospital Anxiety and Depression Scale; IRLSSG, International Restless Legs Syndrome Study Group; MIG w/o RLS, migraine without restless legs syndrome; MIG w RLS, migraine with restless legs syndrome; PSQI, Pittsburgh Sleep Quality Index*.

### Group Differences in Whole-Brain FC Assessed Using NBS

**Figure 2:**
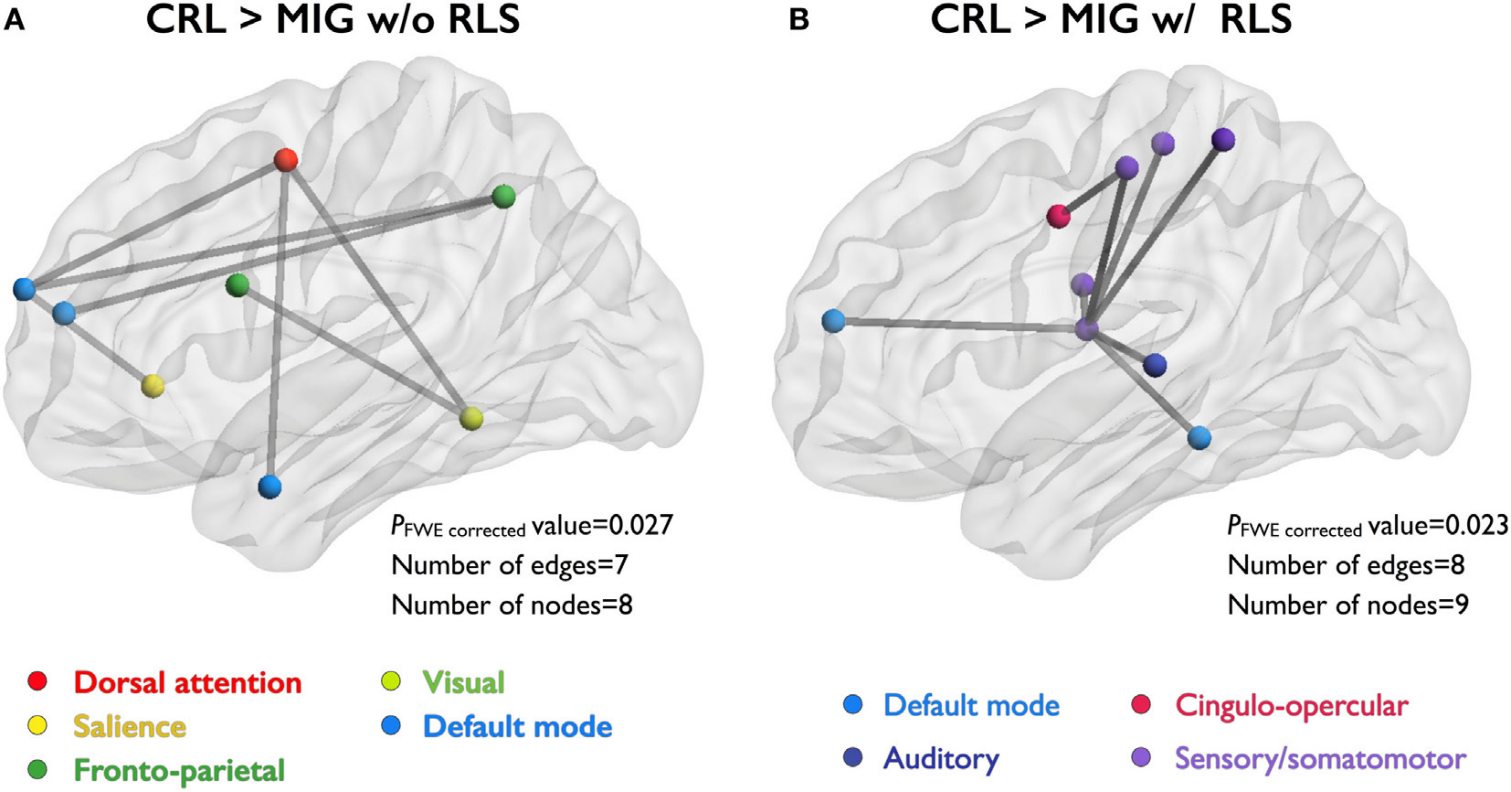
NBS identify the set of abnormal functional connections between study groups. Illustration of the network with reduced FC in two patient groups (compared to CRL group). Node colors correspond with the different functional subsystems. **(A)** Compared to CRL, MIG w/o RLS showed reduced FC in the network with eight nodes and seven edges, and **(B)** MIG w RLS showed reduced FC in the network with nine nodes and eight edges. Abbreviations: CRL, healthy controls; FC, functional connectivity; MIG w/o RLS, migraine without restless legs syndrome; MIG w RLS, migraine with restless legs syndrome; NBS, network-based statistics; FWE, family-wise error.

Network-based statistics analyses revealed that migraine patients without RLS exhibited decreased FC relative to CRL, with eight nodes and seven edges (*P*_FWE-corrected_ = 0.027), in the network composed of the dorsal attention, salience, default mode, visual, and frontoparietal networks (Figure 2A). Migraine patients with RLS exhibited decreased FC relative to CRL, with nine nodes and eight edges (*P*_FWE-corrected_ = 0.023, corrected), in the network composed of the default mode, cingulo-opercular, auditory, and sensory/somatomotor networks (Figure 2B). Importantly, all connections were weaker in patients than controls. However, no differences in disrupted FC components were observed between migraine patients with and those without RLS.

### Group Differences in Network FC between Migraine Patients with and without RLS

**Figure 3:**
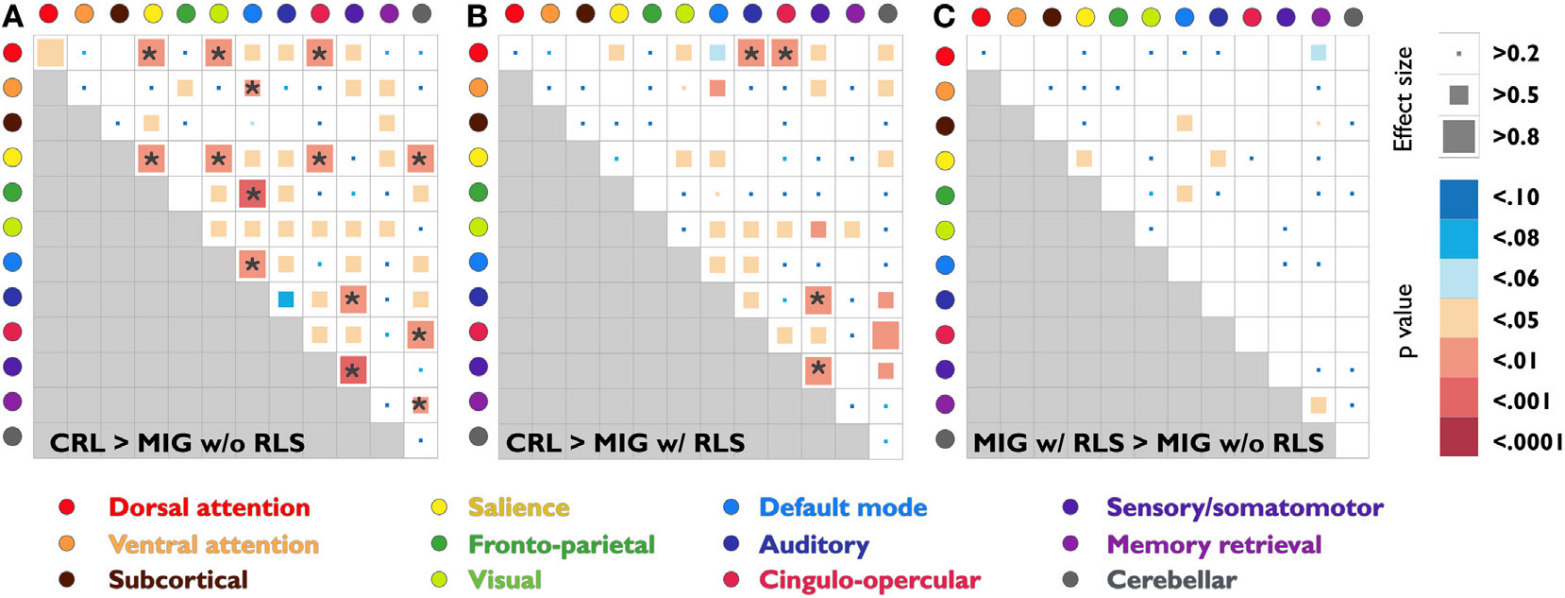
Statistical analysis and effect size estimation of composite *z*-score FC matrices among three study groups **(A)**–**(C)**. Circles with different colors represent 12 functional subsystems. Inside the matrices, the squares with different colors indicate seven statistical levels of matrixwise comparisons between study groups (uncorrected *p*-values); the squares with different sizes indicate the corresponding effect size, which was calculated by Cohen’s *d*. The asterisk indicates that the *p*-value is less than the FDR corrected *p*-value 0.05. Abbreviations: CRL, healthy controls; FC, functional connectivity; FDR, false discovery rate; MIG w/o RLS, migraine without restless legs syndrome; MIG w/RLS, migraine with restless legs syndrome.

Pairwise connectivity analyses revealed that patients with migraine, regardless of RLS diagnosis, exhibited lower FC than CRL in the following networks: salience, default mode, sensory/somatomotor, dorsal attention to salience, visual, auditory and cingulo-opercular, ventral attention to default mode, salience to visual, cingulo-opercular and cerebellar, frontoparietal to default mode, sensory/somatomotor to auditory, cingulo-opercular to cerebellar, and memory retrieval to cerebellar (*P*_FDR-corrected_ < 0.05; Table 2; Figures 3A,B).

**Table 2:** Mean composite *z* scores and statistical results for network pairs between migraine patients with or without RLS and CRL.

Network pair	CRL	MIG	*t*-Value	*p*-Value	Cohen’s *d*
**CRL > MIG w/o RLS**					
DA–SAL	0.20 ± 0.08	0.13 ± 0.06	2.85	0.007	0.88
DA–VIS	0.29 ± 0.10	0.20 ± 0.09	2.99	0.004	0.92
DA–CO	0.29 ± 0.07	0.22 ± 0.09	3.01	0.003	0.89
VA–DM	0.18 ± 010	0.11 ± 0.07	2.40	0.005	0.78
SAL–SAL	0.38 ± 0.10	0.30 ± 0.07	3.13	0.001	0.97
SAL–VIS	0.22 ± 0.08	0.15 ± 0.06	3.05	0.004	0.99
SAL–CO	0.36 ± 0.11	0.29 ± 0.07	2.51	0.006	0.80
SAL–CER	0.22 ± 0.11	0.14 ± 0.06	2.89	0.007	0.91
FP–DM	0.11 ± 0.07	0.04 ± 0.05	3.55	<0.001	1.12
DM–DM	0.30 ± 0.08	0.24 ± 0.08	2.61	0.004	0.85
AU–SEN	0.36 ± 0.16	0.26 ± 0.09	2.69	0.004	0.81
CO–CER	0.32 ± 0.14	0.21 ± 0.11	2.68	0.005	0.85
SEN–SEN	0.44 ± 0.16	0.31 ± 0.11	3.53	<0.001	0.96
MRY–CER	0.10 ± 0.16	0.01 ± 0.13	2.11	0.009	0.64
**CRL > MIG w RLS**					
DA–AU	0.26 ± 0.11	0.17 ± 0.09	3.17	0.002	0.97
DA–CO	0.29 ± 0.07	0.22 ± 0.08	3.13	0.002	0.94
AU–SEN	0.36 ± 0.16	0.25 ± 0.08	2.93	0.002	0.90
SEN–SEN	0.44 ± 0.16	0.33 ± 0.08	3.52	0.001	0.93

*AU, auditory; CER, cerebellar network; CO, cingulo-opercular task control; CRL, healthy controls; DA, dorsal attention; DM, default mode; FP, frontoparietal task control; MIG w/o RLS, migraine without restless legs syndrome; MIG w RLS, migraine with restless legs syndrome; MRY, memory; SAL, salience; SEN, sensory/somatomotor; VA, ventral attention; VIS, visual*.

Further conjunction analyses revealed altered connectivity in the sensory/somatomotor, sensory/somatomotor to auditory, and dorsal attention to cingulo-opercular networks in migraine patients with RLS and without RLS groups. Direct exploratory group comparisons revealed changes in connectivity in the salience, default mode to subcortical and frontoparietal, auditory to salience, and memory retrieval networks between the two patient groups (*P*_uncorrected_ < 0.05; Table 3; Figure 3C).

**Table 3:** Mean composite *z*-scores and statistical results for network pairs between migraine patients with and without RLS.

Network pair	MIG w/RLS	MIG w/o RLS	*t*-Value	*p*-Value	Cohen’s *d*
**MIG w/RLS > MIG w/o RLS**					
SUB–DM	0.07 ± 0.05	0.04 ± 0.05	1.86	0.043	0.56
SAL–SAL	0.34 ± 0.10	0.30 ± 0.07	1.88	0.040	0.50
SAL–AU	0.20 ± 0.11	0.15 ± 0.08	1.85	0.033	0.54
FP–DM	0.08 ± 0.06	0.04 ± 0.05	2.15	0.032	0.65
MRY–MRY	0.46 ± 0.14	0.37 ± 0.15	2.16	0.034	0.64

*AU, auditory; DM, default mode; FP, frontoparietal task control; MIG w/o RLS, migraine without restless legs syndrome; MIG w/RLS, migraine with restless legs syndrome; MRY, memory; SAL, salience; SUB, subcortical*.

### Associations between Clinical Variables and Network Metrics in Migraine Patients with and without RLS

There was a trend for a negative association between cross-network abnormalities in the default mode to subcortical network and RLS severity as assessed using the IRLSSG (*r* = −0.42, *p* = 0.08) in patients with MIG w RLS.

## Discussion

The present study provides evidence for FC alterations in patients with MIG w and w/o RLS. Patients had FC patterns that were different from those of CRL across several networks. These included the dorsal attention, salience, default mode, cingulo-opercular, visual, frontoparietal, auditory, and sensory/somatomotor networks. We also observed similar patterns of FC changes in the sensory/somatomotor, sensory/somatomotor to auditory, and dorsal attention to cingulo-opercular networks in the two patient groups, suggesting that there may be an underlying pathological mechanism common to both groups. Comparison of the two patient groups revealed FC changes in the salience, default mode to subcortical and frontoparietal, auditory to salience, and memory retrieval networks. Finally, we observed trend-level significance for a negative association between RLS severity and cross-network abnormalities of connectivity between the DMN and subcortical network.

In our study, the intra- and interconnectivities of functional networks were significantly lower in patients than in controls, suggesting disruption of functional network organization in both patient groups. Such disruption may result in diffusively impaired functional brain connections. In migraine patients without RLS, the most affected brain functional networks were the dorsal attention, salience, default mode, visual, and frontoparietal networks. Previous studies have demonstrated that dorsal attention network alterations may manifest as memory deficits during and between attacks in patients with migraine (31). Other studies have reported alterations in prefrontal areas associated with attention and the cognitive aspects of pain processing in patients with migraine (14, 32).

Alterations in the DMN have been reported in migraine patients in a number of fMRI studies (14, 33). The DMN includes the precuneus, posterior cingulate cortex, medial prefrontal cortex, medial temporal lobe, and angular gyrus, which are connected and active when a person is at wakeful rest and not performing an attention-demanding task. Alterations in the DMN may be related to dysfunction in the integration of and attention to pain processing in migraine patients (14, 33). The salience network—a core resting-state network that includes the paralimbic–limbic regions—may be involved in the detection of external stimuli and the assignment of attentional resources. FC changes in this network have been reported in fMRI studies of migraine patients without aura (14, 34). In the present study, half of the patients with MIG w/o RLS experienced migraine auras, while previous studies have reported altered FC between the salience and visual networks in such patients (32). These findings may partially explain the observed functional alterations in the visual networks. Previous imaging studies have demonstrated decreased frontoparietal gray matter and frontoparietal network FC in migraine patients and that such changes are associated with attention-related task performance (35). There may thus be a relationship between attention and the frontoparietal network in patients with migraine. Collectively, the involvement of the aforementioned brain networks in patients with MIG w/o RLD is consistent with the reported changes in attention, cognitive function, and pain processing in patients with migraine.

We also observed that migraine patients with RLS exhibited decreased FC in the default mode, cingulo-opercular, auditory, and sensory/somatomotor networks relative to CRL. Functional changes in the DMN have been reported in previous fMRI studies, even during symptom-free periods. These changes may result in difficulties in maintaining a resting state and may involve internal stimuli that urge the patient to move his or her legs while at rest (18). Previous brain imaging studies have indicated that structural and functional changes in the sensorimotor network may be associated with sensory and motor symptoms of the legs in RLS (36, 37).

The cingulo-opercular network is composed of the anterior insula/operculum, anterior cingulate cortex, and thalamus and may be involved in the maintenance of alertness, attentional control, and sensory integration of internal and external stimuli (38). Previous PET studies have also observed altered dopaminergic and opioid activity in brain structures that serve the medial nociceptive system. These structures include the thalamus, anterior cingulate cortex, and insula, which may be involved in the affective-motivational component of pain in patients with RLS (39, 40). A previous neurophysiologic study documented changes in auditory startle responses in patients with RLS, suggesting that the disinhibition of reticulospinal pathways—which likely originate from dysfunctional rostral regions and project to the lower brainstem—is involved in the pathogenesis of RLS (41). Collectively, we observed alterations in these functional networks in patients with MIG w RLS. These changes may be associated with disruptions in attentional control and the management of sensory input information. Our results thus support the hypothesis that RLS is a disorder of somatosensory processing.

Conjunction analysis in both patient groups indicated common patterns of decreased FC relative to control participants in the sensory/somatomotor, sensory/somatomotor to auditory, and dorsal attention to cingulo-opercular networks. These observations indicate that patients with migraine exhibit decreased FC in networks associated with sensory regulation and attention. Indeed, previous studies have revealed that the sensory/somatomotor network in particular is crucial in sensory and pain processing in patients with migraine (42). We also observed that FC between the sensory/somatomotor to auditory and dorsal attention to cingulo-opercular networks was decreased relative to that of controls in both patient groups. Patients with migraine are hypersensitive to sensory stimuli, both during and between attacks (43, 44). Previous physiologic studies on interictal migraine patients indicate the presence of persistent hypersensitivity in the form of lower auditory discomfort thresholds and lower pain thresholds when compared with CRL (45, 46). Habituation deficits and exacerbated attention orientation to auditory event-related potentials have also been reported in patients with migraine, suggesting the abnormal involvement of attention-related networks (47, 48). Additional studies have revealed that auditory startle responses are altered in patients with RLS (41). Our results may partially support the shared clinical characteristic of hypersensitivity to sensory and auditory stimuli in patients with migraine and RLS (41, 46).

In the present study, we observed differences in FC between the two patient groups (migraine patients with and without RLS) with regard to the salience, default mode to subcortical and frontoparietal, salience to auditory, and memory retrieval networks. Most of the observed changes were associated with attentional modulation, sensory, anti-nociception, and limbic networks. Furthermore, it has been reported that children with both migraine and periodic limb movement disorders (including RLS) may have more severe migraine clinical presentation, severity, frequency, and other disabling aspects than children with migraine without periodic limb movement disorder (6). Therefore, these functional differences between the two patient groups may be associated with altered attentional nociceptive control of sensory inputs, which may be responsible for the clinical differences between the two groups, and the survival of undesirable sensations and abnormal sensory-motor integration in patients with RLS (14, 18). Future research is warranted to examine this speculation.

Furthermore, among the patients with RLS, the IRLSSG severity score exhibited a trend-level significance for a negative association with cross-network abnormalities between the default mode and subcortical networks. Weaker connection between these two networks may be associated with higher RLS severity. Migraine is phenotypically different from RLS, as it is characterized by a prominent worsening of symptoms during physical movement. As a result, patients with migraine avoid movement during attacks. In contrast, motor restlessness can only be relieved by voluntary movement in patients with RLS (23). Such findings indicate that RLS may be associated with cortical and subcortical functional connectome anomalies in patients with migraine. Although differences between the two groups in this study did not survive corrections for multiple comparisons, the effect sizes were medium to large, suggesting that our preliminary/exploratory findings can be used to guide future large-scale investigations.

In the present study, we identified the patterns of FC changes in patients with MIG w and w/o RLS. Our findings may aid in the development of neuroimaging-driven biomarkers in future studies. However, the present study possesses some limitations of note. First, the data in this study were obtained using a cross-sectional design. Thus, further longitudinal studies are required to determine whether the functional alterations are dynamic. Second, since most brain networks exhibiting an alteration in the present study are associated with higher cortical function, future studies should employ comprehensive cognitive testing to evaluate the relationships between these brain network alterations and cognition. Third, our sample size was not very large; however, we used a strict definition for diagnosis to ensure high quality of our investigation. Fourth, migraine prevalence peaks between the ages of 20 and 40 years, whereas RLS prevalence peaks after the age of 50 years (1, 5, 10). Furthermore, previous studies have reported that patients with RLS were older than those with comorbid migraine and RLS (49). Therefore, it is difficult to recruit a group of patients with only RLS whose mean age matches to that of migraine patients with RLS. Future studies should examine differences in FC by including an additional group of patients with RLS only. Fifth, to date, no fMRI-detectable brain activity changes have been reported due to the usage of flunarizine, as a preventive agent of migraine. However, topiramate- and propranolol-related differences in blood oxygenated level-dependent signals in fMRI have been reported in epilepsy (50) and autism (51) patients, respectively. Although the patients were asked to stop taking migraine-preventive medications (propranolol, topiramate, or flunarizine) at least 2 days prior to imaging in this study, we cannot fully exclude the potential impact of preventive medications on our findings. Finally, FC analysis does not specify the direction of information propagation in the underlying connected networks, and the contributions of excitatory vs. inhibitory neuronal coupling are unclear (52). We thus cannot determine whether these alterations are part of the pathogenesis or a consequence of the disorders. Future studies involving causality analyses are thus warranted.

In conclusion, we observed disrupted FC in patients with MIG w and w/o RLS when compared to CRL. Both groups displayed a common pattern of FC changes that predominantly affected sensorimotor and attention-related networks. The two groups exhibited FC changes most prominently in the attentional nociceptive control and sensory-related networks. Certain functional network metrics also exhibited a trend-level significance for correlation with RLS severity. Thus, our findings may aid in the development of potential connectome-based biomarkers for MIG w and w/o RLS.

## Ethics Statement

The present study was approved by the Ethics Committee of TVGH and TSGH in Taiwan. All methods were carried out in accordance with the approved guidelines. All participants signed a written informed consent form after a full written and verbal explanation of the study.

## Author Contributions

F-CY, K-HC, A-LH, and SJW participated in data collection, analyzed the data, and drafted the manuscript. F-CY, K-HC, A-LH, J-LF, J-FL, H-WK, C-PL, and S-JW participated in the study design, collected the data, and helped to draft the manuscript. C-PL and S-JW supervised the study, conceptualized and designed the study, and helped to draft the manuscript. All authors read and approved the final manuscript.

## Conflict of Interest Statement

The authors declare that the research was conducted in the absence of any commercial or financial relationships that could be construed as a potential conflict of interest.

## Footnotes

^1^http://afni.nimh.nih.gov/afni/.

^2^http://fsl.fmrib.ox.ac.uk/fsl/fslwiki/.

^3^http://www.fil.ion.ucl.ac.uk/spm.

## Supplementary Material

Figure S1 Illustration of the network with reduced functional connectivity in two disease groups (compared to health control group) under different initial threshold settings with NBS analyses. Abbreviations: CRL, healthy controls; FWE, family-wise error; MIG w/o RLS, migraine without restless legs syndrome; MIG w RLS, migraine with restless legs syndrome; NBS, network-based statistics.
