# Supplementary figures and images for: Altered Brain Functional Connectome in Migraine with and without Restless Legs Syndrome: A Resting-State Functional MRI Study
Source: Front Neurol. 2018 Jan 30;9:25. doi: 10.3389/fneur.2018.00025 (PMC5797592; doi:10.3389/fneur.2018.00025)

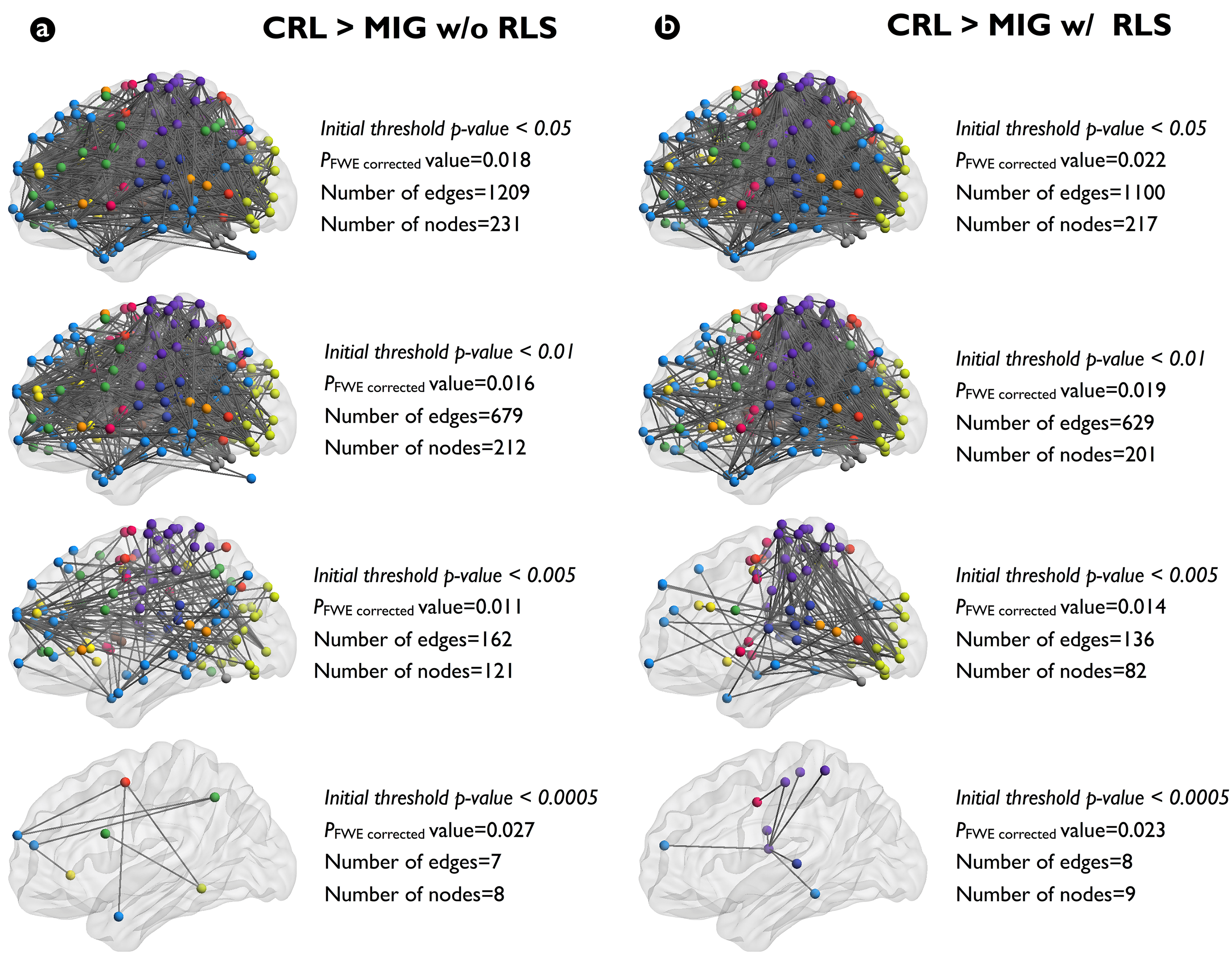

Supplement: Figure S1 — Illustration of the network with reduced functional connectivity in two disease groups (compared to health control group) under different initial threshold settings with NBS analyses. Abbreviations: CRL, healthy controls; FWE, family-wise error; MIG w/o RLS, migraine without restless legs syndrome; MIG w RLS, migraine with restless legs syndrome; NBS, network-based statistics. [file image_1.tif]
