# Supplementary material for: Altered Brain Functional Connectome in Migraine with and without Restless Legs Syndrome: A Resting-State Functional MRI Study
Source: Front Neurol. 2018 Jan 30;9:25. doi: 10.3389/fneur.2018.00025 (PMC5797592; doi:10.3389/fneur.2018.00025)
Supplement: Supplementary file 2 [file table_1.doc]

| **Supplementary Table 1 MNI Coordinates for 236 ROIs of Power’s template.** | | | | | |
| --- | --- | --- | --- | --- | --- |
| ROI | MNI Coordinate | | | Code | Suggested System |
|  | X | Y | Z |  |  |
| 1 | 10 | -62 | 61 | 1 | Dorsal attention |
| 2 | -52 | -63 | 5 | 1 | Dorsal attention |
| 3 | 22 | -65 | 48 | 1 | Dorsal attention |
| 4 | 46 | -59 | 4 | 1 | Dorsal attention |
| 5 | 25 | -58 | 60 | 1 | Dorsal attention |
| 6 | -33 | -46 | 47 | 1 | Dorsal attention |
| 7 | -27 | -71 | 37 | 1 | Dorsal attention |
| 8 | -32 | -1 | 54 | 1 | Dorsal attention |
| 9 | -42 | -60 | -9 | 1 | Dorsal attention |
| 10 | -17 | -59 | 64 | 1 | Dorsal attention |
| 11 | 29 | -5 | 54 | 1 | Dorsal attention |
| 12 | -10 | 11 | 67 | 2 | Ventral attention |
| 13 | 54 | -43 | 22 | 2 | Ventral attention |
| 14 | -56 | -50 | 10 | 2 | Ventral attention |
| 15 | -55 | -40 | 14 | 2 | Ventral attention |
| 16 | 52 | -33 | 8 | 2 | Ventral attention |
| 17 | 51 | -29 | -4 | 2 | Ventral attention |
| 18 | 56 | -46 | 11 | 2 | Ventral attention |
| 19 | 53 | 33 | 1 | 2 | Ventral attention |
| 20 | -49 | 25 | -1 | 2 | Ventral attention |
| 21 | 6 | -24 | 0 | 3 | Subcortical |
| 22 | -2 | -13 | 12 | 3 | Subcortical |
| 23 | -10 | -18 | 7 | 3 | Subcortical |
| 24 | 12 | -17 | 8 | 3 | Subcortical |
| 25 | -5 | -28 | -4 | 3 | Subcortical |
| 26 | -22 | 7 | -5 | 3 | Subcortical |
| 27 | -15 | 4 | 8 | 3 | Subcortical |
| 28 | 31 | -14 | 2 | 3 | Subcortical |
| 29 | 23 | 10 | 1 | 3 | Subcortical |
| 30 | 29 | 1 | 4 | 3 | Subcortical |
| 31 | -31 | -11 | 0 | 3 | Subcortical |
| 32 | 15 | 5 | 7 | 3 | Subcortical |
| 33 | 9 | -4 | 6 | 3 | Subcortical |
| 34 | 11 | -39 | 50 | 4 | Salience |
| 35 | 55 | -45 | 37 | 4 | Salience |
| 36 | 42 | 0 | 47 | 4 | Salience |
| 37 | 31 | 33 | 26 | 4 | Salience |
| 38 | 48 | 22 | 10 | 4 | Salience |
| 39 | -35 | 20 | 0 | 4 | Salience |
| 40 | 36 | 22 | 3 | 4 | Salience |
| 41 | 37 | 32 | -2 | 4 | Salience |
| 42 | 34 | 16 | -8 | 4 | Salience |
| 43 | -11 | 26 | 25 | 4 | Salience |
| 44 | -1 | 15 | 44 | 4 | Salience |
| 45 | -28 | 52 | 21 | 4 | Salience |
| 46 | 0 | 30 | 27 | 4 | Salience |
| 47 | 5 | 23 | 37 | 4 | Salience |
| 48 | 10 | 22 | 27 | 4 | Salience |
| 49 | 31 | 56 | 14 | 4 | Salience |
| 50 | 26 | 50 | 27 | 4 | Salience |
| 51 | -39 | 51 | 17 | 4 | Salience |
| 52 | -44 | 2 | 46 | 5 | Fronto-parietal Task Control |
| 53 | 48 | 25 | 27 | 5 | Fronto-parietal Task Control |
| 54 | -47 | 11 | 23 | 5 | Fronto-parietal Task Control |
| 55 | -53 | -49 | 43 | 5 | Fronto-parietal Task Control |
| 56 | -23 | 11 | 64 | 5 | Fronto-parietal Task Control |
| 57 | 58 | -53 | -14 | 5 | Fronto-parietal Task Control |
| 58 | 24 | 45 | -15 | 5 | Fronto-parietal Task Control |
| 59 | 34 | 54 | -13 | 5 | Fronto-parietal Task Control |
| 60 | 47 | 10 | 33 | 5 | Fronto-parietal Task Control |
| 61 | -41 | 6 | 33 | 5 | Fronto-parietal Task Control |
| 62 | -42 | 38 | 21 | 5 | Fronto-parietal Task Control |
| 63 | 38 | 43 | 15 | 5 | Fronto-parietal Task Control |
| 64 | 49 | -42 | 45 | 5 | Fronto-parietal Task Control |
| 65 | -28 | -58 | 48 | 5 | Fronto-parietal Task Control |
| 66 | 44 | -53 | 47 | 5 | Fronto-parietal Task Control |
| 67 | 32 | 14 | 56 | 5 | Fronto-parietal Task Control |
| 68 | 37 | -65 | 40 | 5 | Fronto-parietal Task Control |
| 69 | -42 | -55 | 45 | 5 | Fronto-parietal Task Control |
| 70 | 40 | 18 | 40 | 5 | Fronto-parietal Task Control |
| 71 | -34 | 55 | 4 | 5 | Fronto-parietal Task Control |
| 72 | -42 | 45 | -2 | 5 | Fronto-parietal Task Control |
| 73 | 33 | -53 | 44 | 5 | Fronto-parietal Task Control |
| 74 | 43 | 49 | -2 | 5 | Fronto-parietal Task Control |
| 75 | -42 | 25 | 30 | 5 | Fronto-parietal Task Control |
| 76 | -3 | 26 | 44 | 5 | Fronto-parietal Task Control |
| 77 | 18 | -47 | -10 | 6 | Visual |
| 78 | 40 | -72 | 14 | 6 | Visual |
| 79 | 8 | -72 | 11 | 6 | Visual |
| 80 | -8 | -81 | 7 | 6 | Visual |
| 81 | -28 | -79 | 19 | 6 | Visual |
| 82 | 20 | -66 | 2 | 6 | Visual |
| 83 | -24 | -91 | 19 | 6 | Visual |
| 84 | 27 | -59 | -9 | 6 | Visual |
| 85 | -15 | -72 | -8 | 6 | Visual |
| 86 | -18 | -68 | 5 | 6 | Visual |
| 87 | 43 | -78 | -12 | 6 | Visual |
| 88 | -47 | -76 | -10 | 6 | Visual |
| 89 | -14 | -91 | 31 | 6 | Visual |
| 90 | 15 | -87 | 37 | 6 | Visual |
| 91 | 29 | -77 | 25 | 6 | Visual |
| 92 | 20 | -86 | -2 | 6 | Visual |
| 93 | 15 | -77 | 31 | 6 | Visual |
| 94 | -16 | -52 | -1 | 6 | Visual |
| 95 | 42 | -66 | -8 | 6 | Visual |
| 96 | 24 | -87 | 24 | 6 | Visual |
| 97 | 6 | -72 | 24 | 6 | Visual |
| 98 | -42 | -74 | 0 | 6 | Visual |
| 99 | 26 | -79 | -16 | 6 | Visual |
| 100 | -16 | -77 | 34 | 6 | Visual |
| 101 | -3 | -81 | 21 | 6 | Visual |
| 102 | -40 | -88 | -6 | 6 | Visual |
| 103 | 37 | -84 | 13 | 6 | Visual |
| 104 | 6 | -81 | 6 | 6 | Visual |
| 105 | -26 | -90 | 3 | 6 | Visual |
| 106 | -33 | -79 | -13 | 6 | Visual |
| 107 | 37 | -81 | 1 | 6 | Visual |
| 108 | -41 | -75 | 26 | 7 | Default mode network |
| 109 | 6 | 67 | -4 | 7 | Default mode network |
| 110 | 8 | 48 | -15 | 7 | Default mode network |
| 111 | -13 | -40 | 1 | 7 | Default mode network |
| 112 | -18 | 63 | -9 | 7 | Default mode network |
| 113 | -46 | -61 | 21 | 7 | Default mode network |
| 114 | 43 | -72 | 28 | 7 | Default mode network |
| 115 | -44 | 12 | -34 | 7 | Default mode network |
| 116 | 46 | 16 | -30 | 7 | Default mode network |
| 117 | -68 | -23 | -16 | 7 | Default mode network |
| 118 | -44 | -65 | 35 | 7 | Default mode network |
| 119 | -39 | -75 | 44 | 7 | Default mode network |
| 120 | -7 | -55 | 27 | 7 | Default mode network |
| 121 | 6 | -59 | 35 | 7 | Default mode network |
| 122 | -11 | -56 | 16 | 7 | Default mode network |
| 123 | -3 | -49 | 13 | 7 | Default mode network |
| 124 | 8 | -48 | 31 | 7 | Default mode network |
| 125 | 15 | -63 | 26 | 7 | Default mode network |
| 126 | -2 | -37 | 44 | 7 | Default mode network |
| 127 | 11 | -54 | 17 | 7 | Default mode network |
| 128 | 52 | -59 | 36 | 7 | Default mode network |
| 129 | 23 | 33 | 48 | 7 | Default mode network |
| 130 | -10 | 39 | 52 | 7 | Default mode network |
| 131 | -16 | 29 | 53 | 7 | Default mode network |
| 132 | -35 | 20 | 51 | 7 | Default mode network |
| 133 | 22 | 39 | 39 | 7 | Default mode network |
| 134 | 13 | 55 | 38 | 7 | Default mode network |
| 135 | -10 | 55 | 39 | 7 | Default mode network |
| 136 | -20 | 45 | 39 | 7 | Default mode network |
| 137 | 6 | 54 | 16 | 7 | Default mode network |
| 138 | 6 | 64 | 22 | 7 | Default mode network |
| 139 | -7 | 51 | -1 | 7 | Default mode network |
| 140 | 9 | 54 | 3 | 7 | Default mode network |
| 141 | -3 | 44 | -9 | 7 | Default mode network |
| 142 | 8 | 42 | -5 | 7 | Default mode network |
| 143 | -11 | 45 | 8 | 7 | Default mode network |
| 144 | -2 | 38 | 36 | 7 | Default mode network |
| 145 | -3 | 42 | 16 | 7 | Default mode network |
| 146 | -20 | 64 | 19 | 7 | Default mode network |
| 147 | -8 | 48 | 23 | 7 | Default mode network |
| 148 | 65 | -12 | -19 | 7 | Default mode network |
| 149 | -56 | -13 | -10 | 7 | Default mode network |
| 150 | -58 | -30 | -4 | 7 | Default mode network |
| 151 | 65 | -31 | -9 | 7 | Default mode network |
| 152 | -68 | -41 | -5 | 7 | Default mode network |
| 153 | 13 | 30 | 59 | 7 | Default mode network |
| 154 | 12 | 36 | 20 | 7 | Default mode network |
| 155 | 52 | -2 | -16 | 7 | Default mode network |
| 156 | -26 | -40 | -8 | 7 | Default mode network |
| 157 | 27 | -37 | -13 | 7 | Default mode network |
| 158 | -34 | -38 | -16 | 7 | Default mode network |
| 159 | 28 | -77 | -32 | 7 | Default mode network |
| 160 | 52 | 7 | -30 | 7 | Default mode network |
| 161 | -53 | 3 | -27 | 7 | Default mode network |
| 162 | 47 | -50 | 29 | 7 | Default mode network |
| 163 | -49 | -42 | 1 | 7 | Default mode network |
| 164 | -46 | 31 | -13 | 7 | Default mode network |
| 165 | 49 | 35 | -12 | 7 | Default mode network |
| 166 | 32 | -26 | 13 | 8 | Auditory |
| 167 | 65 | -33 | 20 | 8 | Auditory |
| 168 | 58 | -16 | 7 | 8 | Auditory |
| 169 | -38 | -33 | 17 | 8 | Auditory |
| 170 | -60 | -25 | 14 | 8 | Auditory |
| 171 | -49 | -26 | 5 | 8 | Auditory |
| 172 | 43 | -23 | 20 | 8 | Auditory |
| 173 | -50 | -34 | 26 | 8 | Auditory |
| 174 | -53 | -22 | 23 | 8 | Auditory |
| 175 | -55 | -9 | 12 | 8 | Auditory |
| 176 | 56 | -5 | 13 | 8 | Auditory |
| 177 | 59 | -17 | 29 | 8 | Auditory |
| 178 | -30 | -27 | 12 | 8 | Auditory |
| 179 | -3 | 2 | 53 | 9 | Cingulo-opercular Task Control |
| 180 | 54 | -28 | 34 | 9 | Cingulo-opercular Task Control |
| 181 | 19 | -8 | 64 | 9 | Cingulo-opercular Task Control |
| 182 | -16 | -5 | 71 | 9 | Cingulo-opercular Task Control |
| 183 | -10 | -2 | 42 | 9 | Cingulo-opercular Task Control |
| 184 | 37 | 1 | -4 | 9 | Cingulo-opercular Task Control |
| 185 | 13 | -1 | 70 | 9 | Cingulo-opercular Task Control |
| 186 | 7 | 8 | 51 | 9 | Cingulo-opercular Task Control |
| 187 | -45 | 0 | 9 | 9 | Cingulo-opercular Task Control |
| 188 | 49 | 8 | -1 | 9 | Cingulo-opercular Task Control |
| 189 | -34 | 3 | 4 | 9 | Cingulo-opercular Task Control |
| 190 | -51 | 8 | -2 | 9 | Cingulo-opercular Task Control |
| 191 | -5 | 18 | 34 | 9 | Cingulo-opercular Task Control |
| 192 | 36 | 10 | 1 | 9 | Cingulo-opercular Task Control |
| 193 | -7 | -52 | 61 | 10 | Sensory/somatomotor Hand or Mouth |
| 194 | -14 | -18 | 40 | 10 | Sensory/somatomotor Hand or Mouth |
| 195 | 0 | -15 | 47 | 10 | Sensory/somatomotor Hand or Mouth |
| 196 | 10 | -2 | 45 | 10 | Sensory/somatomotor Hand or Mouth |
| 197 | -7 | -21 | 65 | 10 | Sensory/somatomotor Hand or Mouth |
| 198 | -7 | -33 | 72 | 10 | Sensory/somatomotor Hand or Mouth |
| 199 | 13 | -33 | 75 | 10 | Sensory/somatomotor Hand or Mouth |
| 200 | -54 | -23 | 43 | 10 | Sensory/somatomotor Hand or Mouth |
| 201 | 29 | -17 | 71 | 10 | Sensory/somatomotor Hand or Mouth |
| 202 | 10 | -46 | 73 | 10 | Sensory/somatomotor Hand or Mouth |
| 203 | -23 | -30 | 72 | 10 | Sensory/somatomotor Hand or Mouth |
| 204 | -40 | -19 | 54 | 10 | Sensory/somatomotor Hand or Mouth |
| 205 | 29 | -39 | 59 | 10 | Sensory/somatomotor Hand or Mouth |
| 206 | 50 | -20 | 42 | 10 | Sensory/somatomotor Hand or Mouth |
| 207 | -38 | -27 | 69 | 10 | Sensory/somatomotor Hand or Mouth |
| 208 | 20 | -29 | 60 | 10 | Sensory/somatomotor Hand or Mouth |
| 209 | 44 | -8 | 57 | 10 | Sensory/somatomotor Hand or Mouth |
| 210 | -29 | -43 | 61 | 10 | Sensory/somatomotor Hand or Mouth |
| 211 | 10 | -17 | 74 | 10 | Sensory/somatomotor Hand or Mouth |
| 212 | 22 | -42 | 69 | 10 | Sensory/somatomotor Hand or Mouth |
| 213 | -45 | -32 | 47 | 10 | Sensory/somatomotor Hand or Mouth |
| 214 | -21 | -31 | 61 | 10 | Sensory/somatomotor Hand or Mouth |
| 215 | -13 | -17 | 75 | 10 | Sensory/somatomotor Hand or Mouth |
| 216 | 42 | -20 | 55 | 10 | Sensory/somatomotor Hand or Mouth |
| 217 | -38 | -15 | 69 | 10 | Sensory/somatomotor Hand or Mouth |
| 218 | -16 | -46 | 73 | 10 | Sensory/somatomotor Hand or Mouth |
| 219 | 2 | -28 | 60 | 10 | Sensory/somatomotor Hand or Mouth |
| 220 | 3 | -17 | 58 | 10 | Sensory/somatomotor Hand or Mouth |
| 221 | 38 | -17 | 45 | 10 | Sensory/somatomotor Hand or Mouth |
| 222 | -49 | -11 | 35 | 10 | Sensory/somatomotor Hand or Mouth |
| 223 | 36 | -9 | 14 | 10 | Sensory/somatomotor Hand or Mouth |
| 224 | 51 | -6 | 32 | 10 | Sensory/somatomotor Hand or Mouth |
| 225 | -53 | -10 | 24 | 10 | Sensory/somatomotor Hand or Mouth |
| 226 | 66 | -8 | 25 | 10 | Sensory/somatomotor Hand or Mouth |
| 227 | 47 | -30 | 49 | 10 | Sensory/somatomotor Hand or Mouth |
| 228 | -2 | -35 | 31 | 11 | Memory |
| 229 | -7 | -71 | 42 | 11 | Memory |
| 230 | 11 | -66 | 42 | 11 | Memory |
| 231 | 4 | -48 | 51 | 11 | Memory |
| 232 | 2 | -24 | 30 | 11 | Memory |
| 233 | -16 | -65 | -20 | 12 | Cerebellar |
| 234 | -32 | -55 | -25 | 12 | Cerebellar |
| 235 | 22 | -58 | -23 | 12 | Cerebellar |
| 236 | 1 | -62 | -18 | 12 | Cerebellar |

Abbreviations: MNI, Montreal Neurological Institute; ROIs, region of interest.
